# Unveiling Small Non‐Coding RNA Dynamics During Recombinant Adeno‐Associated Virus Production

**DOI:** 10.1002/biot.70092

**Published:** 2025-08-06

**Authors:** Madina Burkhart, Katrin Langenbach, Karlheinz Holzmann, Nadine Hornung, Jamie‐Ann Baiz, Kerstin Otte

**Affiliations:** ^1^ Ulm University Helmholtzstr. 16, Ulm Germany; ^2^ Institute for Applied Biotechnology University of Applied Sciences Biberach Biberach Germany; ^3^ Core Facility Genomics Center for Biomedical Research University Hospital Ulm Ulm Germany

**Keywords:** HEK293F, manufacturing, microRNA, production, rAAV, snoRNA

## Abstract

Recombinant adeno‐associated viruses (rAAVs) play a pivotal role in gene therapy, yet the molecular interactions underlying rAAV production in host cells remain incompletely understood. Non‐coding RNAs (ncRNAs), particularly microRNAs (miRNAs) and small nucleolar RNAs (snoRNAs), are increasingly recognized as key regulators of viral and cellular processes. This study investigates the dynamic expression profiles of miRNAs and snoRNAs during rAAV plasmid transfection and vector production in HEK293F cells. A total of 142 miRNAs were differentially expressed during the peak phase of rAAV production, with 128 associated with the Gene Ontology term “viral process”, indicating broad involvement in host‐virus interactions. Target gene analysis linked these miRNAs to biological pathways such as nucleocytoplasmic transport, innate immunity, apoptosis, and transcriptional regulation, highlighting potential roles of miRNAs in shaping the cellular environment during viral vector assembly. In contrast, snoRNAs exhibited more modest changes in expression, yet five were significantly differentially expressed during active production, suggesting a possible, underexplored involvement in viral replication. These findings illuminate the underexplored contributions of ncRNAs to the host response during rAAV biogenesis and provide a valuable resource for understanding how cellular regulatory networks are engaged throughout vector production.

## Graphical Abstract and Lay Summary

In this study, we explored the largely underexamined role of non‐coding RNAs in recombinant adeno‐associated virus (rAAV) production. Using a time‐course, multi‐layered analytical approach in HEK293F cells, we identified 142 differentially expressed microRNAs and five snoRNAs involved in key cellular processes, including nucleocytoplasmic transport, immune response, and transcriptional regulation. These findings provide new insights into the molecular landscape of rAAV biogenesis and highlight previously unrecognized regulatory layers that may suggest potential strategies for improving vector manufacturing.

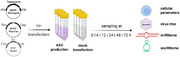

**Abbreviations**
AAV: Adeno‐associated virus
BCL2: BCL2 Apoptosis Regulator
cAMP: Cathelicidin Antimicrobial Peptide
CGAS: cyclic GMP‐AMP synthase
DAVID: database for annotation, visualization and integrated discovery
DPGP: Dirichlet process Gaussian mixture model
EIF2AK2: eukaryotic translation initiation factor 2 alpha kinase 2
EP300: E1A binding protein P300
FC: fold change
GFP: green fluorescent protein
IKBKB: inhibitor of nuclear factor kappa B kinase subunit beta
IRF: interferon regulatory factor
JAK1: Janus Kinase 1
HEK293: human embryonic kidney 293 cells
hpT: hours post‐transfection
Huh7: human hepatocellular carcinoma cells
MAVS: mitochondrial antiviral signaling protein
miRNA: microRNA
Mre11: MRE11 double strand break repair nuclease
MRN: Mre11‐Rad50‐Nbs1 complex
ncRNA: non‐coding RNA
NUP: nucleoporin
PEI: polythyleneimine
PHF5A: PHD finger‐like domain protein 5A
PKA: protein Kinase A
PKR: protein Kinase R
PML: PML nuclear body scaffold
rAAV: recombinant adeno‐associated virus
VCD: viable cell density
SEAP: secreted embryonic alkaline phosphatase
snoRNA: small nucleolar RNA
snoRNP: small nucleolar ribonucleoprotein
SNW1: SNW Domain Containing 1
SMARCB1: SWI/SNF related BAF chromatin remodeling complex subunit B1
SP1: SP1 transcription factor
STAT1: signal transducer and activator of transcription 1
STING1: stimulator of interferon response CGAMP interactor 1
TICAM1: TIR domain containing adaptor molecule 1
UT: untransfected control

## 1 Introduction

Recombinant adeno‐associated viruses (rAAVs) are pivotal tools in gene therapy, offering high specificity, stable episomal transgene expression, and low immunogenicity [1]. Their clinical success includes approved therapies for inherited retinal diseases and spinal muscular atrophy, positioning rAAVs at the forefront of genetic medicine [2]. However, scalable production remains a critical bottleneck due to low yields, high costs, and batch variability, hindering the growing clinical and commercial demand [3, 4]. These challenges underscore the need to better understand the biological mechanisms underlying vector production, particularly the cellular responses to the stresses imposed during rAAV manufacturing.

Efficient rAAV production is shaped by the host cell biology intricating processes like viral replication, capsid assembly, and host‐virus interactions [5, 6]. Moreover, mammalian cell lines such as HEK293 possess innate sensing mechanisms for exogenous DNA of both bacterial and viral origin, which may impact the cell's capacity for rAAV production [7]. In addition, triple plasmid transfection and the expression of specific viral elements, including viral genomes and adenoviral helper genes, have been shown to induce distinct stress responses in host cells that were not observed following transfection with empty plasmids or treatment with the transfection reagent polyethyleneimine (PEI) alone [8]. A prominent example of cytotoxicity mediated via viral protein expression was reported for the large Rep78 protein capable of inducing apoptosis as described by Schmidt and colleagues [9].

Furthermore, the adenoviral E1A protein, a universal viral expression modulator, interacts with host cell components of the Cathelicidin Antimicrobial Peptide/Protein Kinase A (cAMP/PKA) signaling axis, which in turn directly controls the adenoviral *E2* gene complex encoding for essential proteins for viral DNA replication [10, 11]. Additionally, the *E1B* gene encodes oncoproteins that suppress E1A‐induced apoptosis and inhibit the tumor suppressor p53, collectively facilitating cell cycle progression and suppressing apoptotic pathways [12]. Moreover, the interaction between E4 and E1B proteins attenuates the host cell DNA damage response by interfering with Mre11, a double strand break repair nuclease, thereby disrupting the cellular Mre1‐Rad50‐Nbs1 repair complex (MRN) and impairing genome surveillance mechanisms [13].

Small non‐coding RNAs (ncRNAs), including microRNAs (miRNAs) and small nucleolar RNAs (snoRNAs), have emerged as potential regulators during viral infection [14, 15, 16, 17, 18]. MiRNAs, ∼22 nt in length, post‐transcriptionally regulate gene expression by binding mRNAs to suppress translation or mediate degradation. Furthermore, miRNAs are able to target multiple genes and networks, positioning them as crucial regulators in complex biological systems [19]. SnoRNAs, 60–300 nt long, primarily reside in the nucleus and guide chemical modifications of ribosomal RNAs via snoRNP complexes. Each snoRNA includes antisense elements for RNA targeting and is categorized into C/D box, H/ACA box, and scaRNAs [20, 21]. C/D box snoRNAs mediate 2′‐O‐methylation, influencing RNA stability, structure, and interactions, essential for processes like gene regulation, translation, and immune recognition [22]. H/ACA box snoRNAs facilitate pseudouridylation, stabilizing RNA conformations and modulating RNA‐protein interactions, impacting RNA metabolism and gene expression [23]. ScaRNAs combine C/D and H/ACA box motifs with an additional element for localization in Cajal bodies [24]. Beyond these canonical roles, snoRNAs also interact with mRNAs and non‐canonical proteins, but snoRNAs being involved in viral infections and rAAV production is underexplored [25, 26]. Recent studies suggest that non‐coding RNAs can modulate viral replication, transcriptional regulation, and host defense mechanisms, making them attractive targets for improving rAAV production. For instance, miRNAs have been implicated in the regulation of viral genome processing, capsid protein synthesis, and host cell stress responses, potentially influencing vector yield and quality [27]. Likewise, snoRNAs have been linked to the cellular stress response and host‐virus interactions, processes critical for efficient rAAV replication [16, 18, 28].

While advances in multi‐omics technologies – including transcriptomics, proteomics, and metabolomics – have begun to elucidate key pathways active during rAAV production [29], integrating ncRNA expression profiles into this framework remains a significant gap. Omics‐based investigations have highlighted pathways related to immune activation, nucleocytoplasmic transport, and apoptosis as central to the production environment [5, 30, 31, 32, 33, 34, 35, 36 ], yet the upstream regulators, many of which may include miRNAs and snoRNAs, are not fully defined. By examining non‐coding RNA dynamics alongside these broader molecular changes, researchers can develop a more complete picture of how HEK293 cells interpret and respond to the demands of rAAV production.

To expand this understanding, we conducted a comprehensive miRNome and snoRNome analysis of HEK293 cells during rAAV production – a regulatory layer that has not been systematically analyzed to date. Therefore, HEK293F cells were either mock‐ or rAAV transfected and cultivated for over 72 h. During cultivation and production, samples were taken to analyze cellular parameters and rAAV titers. Ultimately, RNA specimens of each sampling time point were isolated and analyzed via microarray expression analysis. Our findings revealed significant differential expression (difference in fold change ≥1.5, *p* value <0.05; FDR <0.1) of miRNAs and snoRNAs, particularly during the main phase of rAAV production between 24 and 72 hours post‐transfection (hpT). Differentially expressed miRNAs and their regulated target genes were subjected to GO term analysis and revealed biological processes that might affect rAAV production. Our findings provide a foundational framework for future efforts to define the regulatory logic of non‐coding RNAs in rAAV manufacturing environments.

## 2 Material and Methods

### 2.1 Cell Culture

Suspension‐adapted human embryonic kidney cells (HEK293F, Thermo Fisher Scientific; Waltham, USA) were routinely grown in 125 mL Erlenmeyer shake flasks (Corning; New York, USA) and BalanCD HEK293 medium (FUJIFILM Irvine Scientific; Santa Ana, USA) supplemented with 4 mM L‐Glutamine (Thermo Fisher Scientific; Waltham, USA). Viable cell density (VCD) and viability were assessed by trypan blue via CEDEX XS cell counter measurement (Roche Diagnostics; Mannheim, Germany). Cells were maintained at 37°C, 5% CO_2_, and 85% humidity with agitation at 140 rpm (25 mm orbit) (Kuhner; Birsfelden, Switzerland).

Human hepatocellular carcinoma cells (Huh7, kindly provided by Prof. Dr. Stefan Kochanek) were maintained in DMEM low glucose (Th. Geyer GmbH & Co.KG; Renningen, Germany) supplemented with 1% GlutaMAX (Fisher Scientific; Waltham, USA) and 10% FBS (Capricorn Scientific GmbH; Ebsdorfergrund, Germany). Viable cell density (VCD) and viability were assessed using Neubauer counting chamber by means of trypan blue exclusion (VWR; Darmstadt, Germany).

### 2.2 Plasmid Cloning

The pAAV‐GFP plasmid was a gift from John T Gray (Addgene plasmid #32395) and was used as starting plasmid to replace the green fluorescent protein (GFP) insert by secreted embryonic alkaline phosphatase (SEAP) derived from the CMV‐SEAP plasmid, which was a gift from Alan Cochrane (Addgene plasmid #24595). The SEAP insert was amplified via overhang PCR using the following primers: SEAP fw: ATATACCGGTCTGCCCTCCAGACATGCTG 3‘; SEAP rev: ATATAGATCTGGCCAGCAGAGGAAGCAA 3‘. Following that, both pAAV‐GFP vector backbone and amplified insert were cut with restriction enzymes *BglII* and *AgeI* (Thermo Fisher Scientific; Waltham, USA) and ligated using T4 Ligase (1 U/µL) (Thermo Fisher Scientific; Waltham, USA). Transformation was performed in One Shot TOP10 chemically competent *E. coli* (Thermo Fisher Scientific; Waltham, USA) following Midi plasmid preparation according to manufacturer instructions (NucleoBond Xtra Midi Kit, Macherey‐Nagel; Düren, Germany).

### 2.3 rAAV Plasmid Transfection

For rAAV production, pAAV‐SEAP transgene plasmid, rep/cap plasmid pAAV2/2, which was a gift from Melina Fan (Addgene plasmid #104963), and pAdDeltaF6 helper plasmid, which was a gift from James M. Wilson (Addgene plasmid #112867), were mixed in a molar ratio of 1:1:1 to a final concentration of 1 pg DNA per viable cell and transfected using PEIpro (Polyplus; Illkirch, France). In addition, untransfected cells (UT) for growth monitoring and a mock transfection using the same amounts of transfection reagents but lacking plasmid DNA were included. For transfection, HEK293F cells were seeded at 2 × 10^6^ viable cells/mL (vc/mL) in a total culture volume of 10 mL in TubeSpin bioreactor tubes (TPP; Trasadingen, Switzerland). It should be noted that the mock and rAAV transfections were based on the same pre‐transfection starting cell culture. Immediately before transfection (0 h) and 6, 12, 24, 48, and 72 h post‐transfection (hpT), samples were taken for measurement of cellular parameters including VCD and viability as described earlier, titer analysis, and finally, the microarray‐based expression analysis.

### 2.4 Quantification of Vector Genome Titer and Functional Infectivity

For rAAV titer determination, HEK293F cells were freeze‐thawed in three consecutive cycles for cell lysis, followed by centrifugation at 3700 x g for 15 min to release the rAAV particles. Thereof, the encapsidated vector genomes were quantified via qPCR. Ten microliters crude lysate of each sample were DNase I (Thermo Fisher Scientific; Waltham, USA) digested with 10 U per sample and incubated for 1 h at 37°C. The enzymatic reaction was stopped by incubating for 10 min at 75°C. Following that, samples were treated with 10 µL Proteinase K (>600 mAU/mL) (Thermo Fisher Scientific; Waltham, USA), incubated for 2 h at 56°C, and inactivated for 30 min at 95°C. For each sample, a dilution series of 5X to 2500X was performed, whereof 5 µL per sample were applied in triplicates for qPCR quantification using the GreenMasterMix (2X) without ROX (Genaxxon; Ulm, Germany) and 0.15 µL each of forward and reverse primer (100 µM stock solution) (fw: 5’‐ GCCGCCAAGAACCTCATCAT‐3’; rev: 5’‐ GTCCATGGCCAGGGGTATC‐3’). To generate the standard curve for vector genome quantification, linearized plasmid DNA (stock concentration: 2 × 10^8^ molecules/µL) containing the SEAP transgene was serially diluted to a final amount of 1 × 10^8^ to 1 × 10^3^ per well and applied in triplicates. The qPCR was performed using the following parameters: initial denaturation at 95°C for 10 min; denaturation, annealing, and extension at 95°C for 10 s and 58°C for 15 s for in total of 40 cycles.

To determine the infectivity, a receiver cell assay was performed. Therefore, Huh7 cells were seeded at a density of 2.5 × 10^4^ vc/well in Nunc Delta 96‐well flat bottom plates (Thermo Fisher Scientific; Waltham, USA) and incubated for 5 h at 37°C, 5 % CO_2,_ and 85% humidity. After that, cells were treated with 0.15 µg/mL Mitomycin C (Stem Cell Technologies; Cologne, Germany) for 1 h, washed with supplemented DMEM low glucose without FBS, and transduced with 10 µL crude lysate containing rAAV particles, which was sampled over distinct time points. The day after, a medium change using supplemented DMEM low glucose with 5% FBS was performed, and cells were incubated for another 48 h prior to chemiluminescent quantification via Tecan Infinite PRO microplate reader (Tecan Group; Männedorf, Switzerland).

### 2.5 Microarray Analysis

Total RNA and miRNA were isolated in biological triplicates from 5 × 10^5^ viable cells taken from mock‐ and rAAV plasmid‐transfected HEK293F cells over the entire time course of cultivation and production using the miRNeasy Kit for miRNA Purification according to the manufacturer's instructions (Qiagen; Hilden, Germany). RNA quality and integrity were assessed using an Agilent 2100 TapeStation 4200 (Agilent Technologies; Palo Alto, USA). RNA integrity number (RIN) values were≥9 for all samples, indicating a very high RNA quality. Furthermore, RNA purity and concentration were determined by absorbance measurement at 260 and 230 nm via Nanodrop 1000 Spectrophotometer (Thermo Fisher Scientific; Darmstadt, Germany). The GeneChip miRNA 4.0 Arrays (Thermo Fisher Scientific; Darmstadt, Germany) were employed to analyze the differential expression of miRNAs in un‐, mock‐ and rAAV‐transfected cells. Therefore, 200 ng of total RNA was biotin labelled using the FlashTag Biotin HSR RNA Labeling Kits (Thermo Fisher Scientific; Darmstadt, Germany). The arrays were then hybridized, stained, and washed in accordance with the manufacturer's instructions on a GeneChip Fluidics Station 450 (Affymetrix; Santa Clara, USA) and scanned on a GeneChip Scanner 3000G (Affymetrix; Santa Clara, USA). The raw feature data were normalized using the Robust Multiarray Average (RMA) method [37] of the Expression Console software from Affymetrix (Affymetrix; Santa Clara, USA). As the Affymetrix miRNA array contains miRNAs from several species, the normalization was restricted to human probe sets only. This procedure has proven to produce reliable results as published previously [38]. A transcriptome analysis was performed using BRB‐ArrayTools developed by Dr. Richard Simon and BRB‐ArrayTools Development Team (http://linus.nci.nih.gov/BRB‐ArrayTools.html). We identified non‐coding RNAs that were differentially expressed among the classes using a two‐sample *t*‐test. Differential expression was considered statistically significant with a *p* value below 0.05 and a fold change between the two groups of at least 1.5‐fold. We used the Benjamini and Hochberg correction [39] to provide 90% confidence that the false discovery rate was less than 10%. Only probe sets that were present in all three replicates of at least one group, according to the Affymetrix Detection Above Background (DABG) algorithm, were used for differential expression analysis. The data discussed in this publication have been deposited in NCBI's Gene Expression Omnibus [40] and are accessible through GEO Series accession number GSE289823 (https://www.ncbi.nlm.nih.gov/geo/query/acc.cgi?acc = GSE289823).

### 2.6 Data Evaluation and Statistical Analysis

Time‐series analysis was conducted using a Python (version 2.7.18)‐based implementation of a Dirichlet Process Gaussian Process (DPGP) mixture model (https://github.com/PrincetonUniversity/DP_GP_cluster) to cluster miRNAs based on their expression patterns [41]. The DPGP approach assumes that expression levels at neighboring time points are correlated, making this analysis well‐suited for modeling time course‐derived data. Although *z*‐score standardization is commonly used in time‐series clustering to emphasize relative temporal patterns, in this study, we tested both standardized and non‐standardized data but retained relative log2‐transformed expression values without standardization. This approach preserves both the direction and magnitude of expression changes, which were essential for biological interpretation and downstream analyses. Additionally, we included all detected miRNAs in the clustering (*n* = 636), regardless of differential expression status. A substantial proportion of miRNAs were found to be differentially expressed based on two independent comparative strategies – against both mock‐transfected controls and basal expression levels – highlighting the broad regulatory shifts occurring in the dataset. To avoid excluding potentially relevant miRNAs through strict cutoffs, we opted to retain the full set, which enhanced clustering resolution and biological interpretability. The DPGP mixture model is capable of handling real‐valued inputs, and clustering on unstandardized log2 fold changes across all miRNAs yielded trajectories that more accurately reflected the underlying gene regulatory dynamics. Accordingly, relative log2‐transformed fold changes in miRNA expression were submitted to clustering with 1000 iterations of cluster assignment.

Target gene and pathway prediction of miRNAs was performed using DIANA miRPath 4.0 based on the experimentally supported DIANA‐TarBase v8.0 database and the miRTarBase 2022 [42, 43, 44]. Retrieved target genes were further analyzed using Database for Annotation, Visualization and Integrated Discovery (DAVID) [45]. Gene symbols were taken from GeneCards [46]. Statistical analysis was conducted using GraphPad Prism 9 software (GraphPad Software; San Diego, USA). Student's two‐sample *t*‐test was applied to calculate statistical differences of the obtained data. Unless otherwise stated, all data sets are given as biological triplicates (mean ± SD).

## 3 Results

### 3.1 Cellular Parameters and Titer Analysis During rAAV Production

**FIGURE 1:**
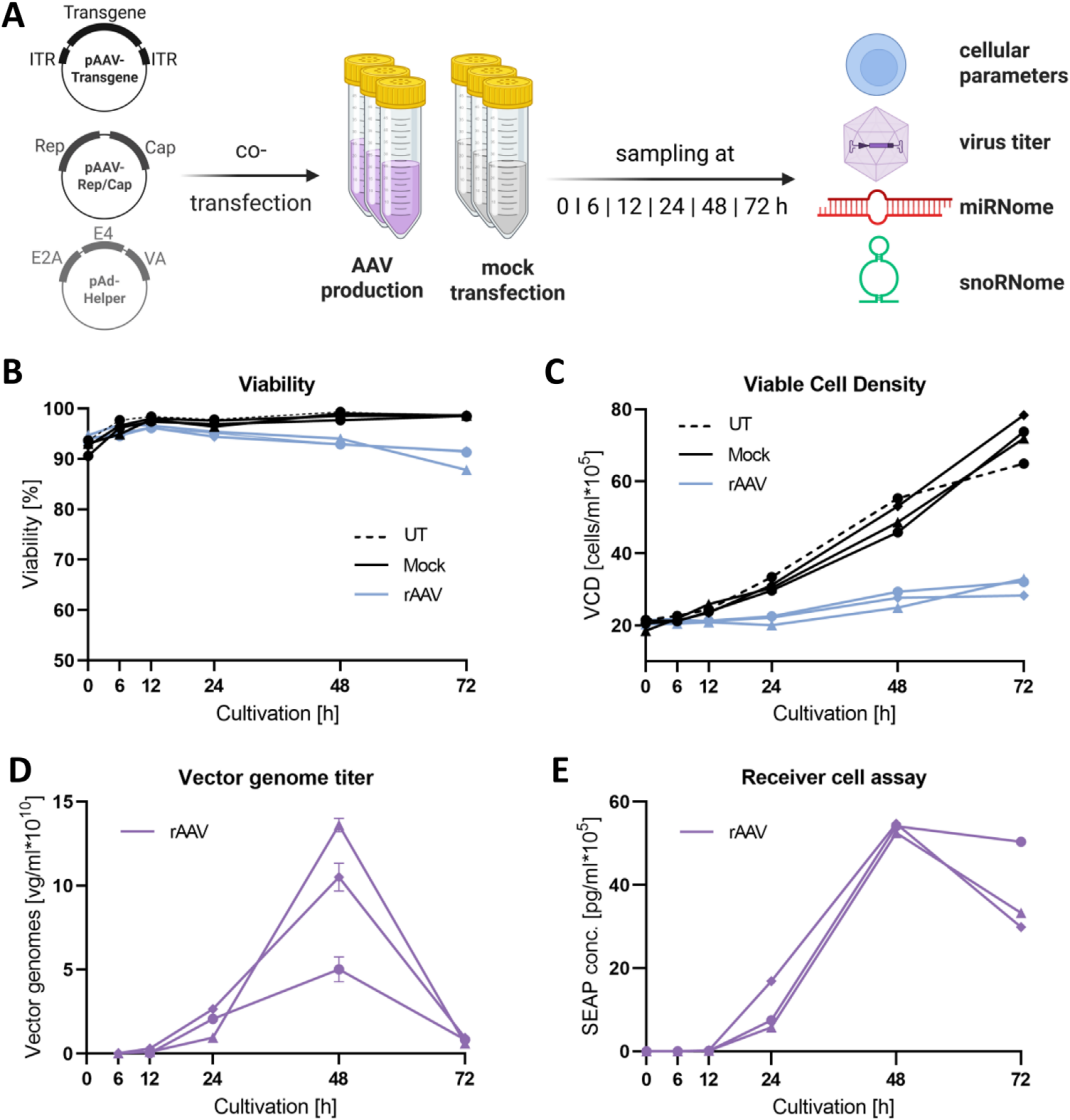
Experimental setup, monitoring of cellular parameters, and titer analysis to investigate expression dynamics of miRNAs and snoRNAs in HEK293F cells during mock‐ and rAAV transfection. (A) Three independent biological replicates for each condition were sampled prior to transfection (0 h), as well as 6, 12, 24, 48, and 72 h post‐transfection (hpT). RNA was prepared from in total 33 samples and subjected to microarray expression analysis. In addition, cellular parameters and titer were monitored over the entire course of cultivation and production. Created with BioRender.com. (B)–(C) Viability and Viable Cell Density (VCD) were determined for mock (black) and rAAV transfection (light blue) in four technical replicates per sample. An untransfected control (UT) was included to monitor growth (black dashed line). (D)–(E) Vector genome titer and infectivity via receiver cell assay were analyzed in biological triplicates via quantification of pAAV‐Transgene‐encoded secreted embryonic alkaline phosphatase (SEAP) either by qPCR using transgene‐specific primer pairs or by transduction of Huh7 cells, followed by a chemiluminescent read‐out.

To investigate the expression dynamics of miRNAs and snoRNAs during rAAV production, a triple rAAV plasmid transfection of HEK293F cells was performed mediated by PEI, as well as a mock transfection lacking rAAV plasmids, each in biological triplicates (Figure 1A). Throughout the course of subsequent cultivation and production, samples were taken directly before transfection at 0 h, as well as at 6, 12, 24, 48, and 72 h post‐transfection (hpT). At each sampling time point, cellular parameters, as well as rAAV vector genome titer and infectivity in a cell receiver assay, were monitored. Finally, the RNA of in total of 33 collected samples was isolated and applied for Chip‐based expression analysis.

After rAAV transfection, high viabilities were observed with a slight decrease to approximately 90% starting at 24 hpT compared to mock transfection and the non‐transfected control (Figure 1B). Similarly, rAAV‐transfected cells displayed slower growth with a final viable cell density (VCD) of only approx. 3 × 10^6^ cells/mL, and thus, VCD did not even double during cultivation (Figure 1C). Both vector genome titer and infectivity started to considerably increase from 24 hpT, with a peak at 48 hpT and a decrease towards 72 hpT, which might correlate with decreased viability during cultivation (Figure 1D,E). The main rAAV production phase was attributed to the later cultivation time points from 24 to 72 hpT.

### 3.2 Differential miRNA Regulation During rAAV Production

**FIGURE 2:**
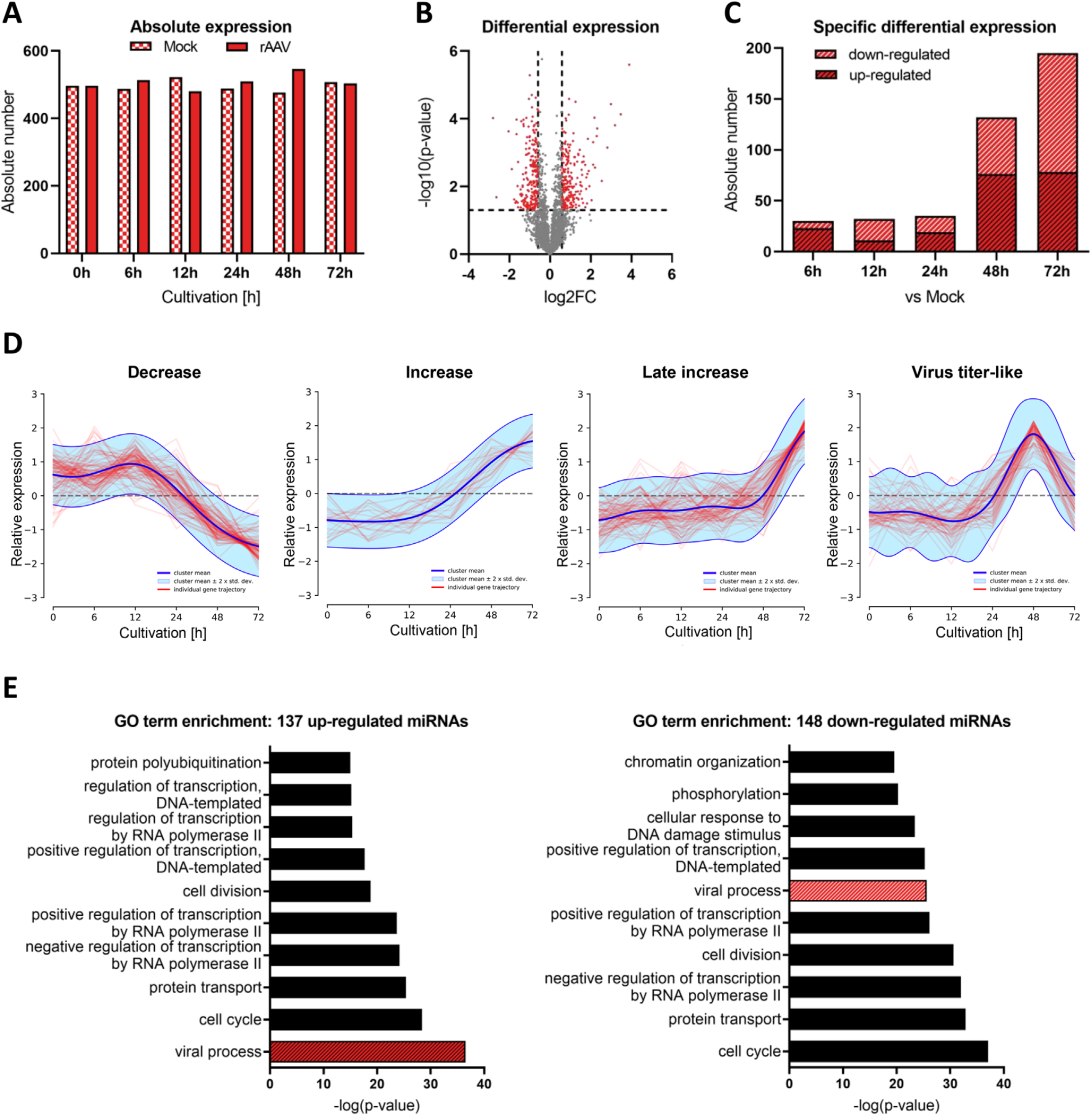
Overview of miRNA expression dynamics during rAAV transfection relative to mock transfection. (A) Absolute number of miRNAs expressed during mock (checked) and rAAV transfection (filled) at each sampling time point. (B) Volcano plot considering all expression events (grey and red dots, *n* = 2708) during rAAV transfection relative to mock transfection. Differential expression is considered significant (red dots, *n* = 426) for a log2 fold change (log2FC) of ±0.5849 (vertical lines) and a ‐log10 *p* value of 1.3 (horizontal line). (C) rAAV‐specific number of up‐ and downregulated miRNAs (difference in fold change ≥1.5, *p* value <0.05; FDR <0.1) at each sampling time point relative to mock transfection. (D) miRNA clustering according to temporal changes during rAAV transfection using the DPGP mixture model. The x and y axis represent sampling time points (in hours) and relative log2‐transformed expression values to mock transfection without standardization for all biological triplicates. Red lines represent individual trajectories for each miRNA relative to mock transfection, while the dark blue line indicates the mean of clustered miRNAs. In addition, the range of 95% confidence interval ±2 SD is given in light blue. **E** GO term enrichment for up‐ and downregulated miRNAs relative to mock transfection and associated experimentally validated target genes during the main rAAV production phase (24–72 hpT) using miRPath 4.0. Term overrepresentation in targets of the input miRNAs were evaluated using one‐side Fisher's exact test. Significant terms in GO domain ‘biological process’ were further filtered using genes union as merging method, a *p* value threshold of <0.05 and false discovery rate (FDR) correction.

To investigate miRNA expression during rAAV production, miRNome analysis was performed in triplicates at all previously described time points for both mock‐ and rAAV‐transfected HEK293F cells. Across the cultivation and production process, a total number of 636 miRNAs were expressed at any sampling time point, with comparable numbers observed in rAAV or mock‐transfected cells (Figure 2A). Notably, the highest number was recorded at 48 hpT in rAAV‐transfected cells, with 546 miRNAs, followed by 522 miRNAs at 12 hpT in mock‐transfected cells.

To identify differentially expressed miRNAs potentially associated with virus production, we implemented a multi‐layered comparison strategy. First, differential miRNA expression was calculated for each time point as log2 fold change of rAAV transfection relative to mock transfection. This analysis revealed in total 424 differential expression events comprising 207 up‐regulation events at any time point with up to 3.9‐fold change and 217 down‐regulation events with up to −2.8‐fold change (Figure 2B). Notably, almost 50% of miRNAs showed differential expression relative to mock at one or more time points (Table S1). The number of differentially expressed miRNAs increased progressively over time, with the most pronounced differences observed at 48 and 72 hpT (Figure 2C). With respect to the temporal expression patterns of individual miRNAs relative to mock transfection, the DPGP analysis identified 18 distinct clusters (Figure S1), whereof four covered 44.5% of all miRNAs. These clusters represented miRNAs with decreasing, increasing, and late increasing patterns, as well as one cluster showing a virus titer‐like expression (Figure 2D) consistent with rAAV vector genomes and infectivity progression shown in Figure 1D,E.

The above‐described analyses revealed that the number of differentially expressed miRNAs increased significantly during the main phase of rAAV production, starting at 24 hpT. This observation suggests that these miRNAs may play a role in, or be influenced by, the molecular events occurring during rAAV production. To explore this further, bioinformatic analysis of all 285 miRNAs differentially regulated between 24 and 72 hpT was performed (Table S2A). Using DIANA‐miRPath v4.0 [42], we analyzed experimentally validated target genes of differentially regulated miRNAs and conducted a GO term enrichment analysis for biological processes. The GO term “viral process” was found to be highly significantly enriched for both up‐regulated and down‐regulated miRNAs, alongside terms related to transcription regulation, cell cycle, and cell maintenance (Figure 2E). These findings strongly suggest that differentially expressed miRNAs are involved in the molecular changes associated with rAAV production.

**FIGURE 3:**
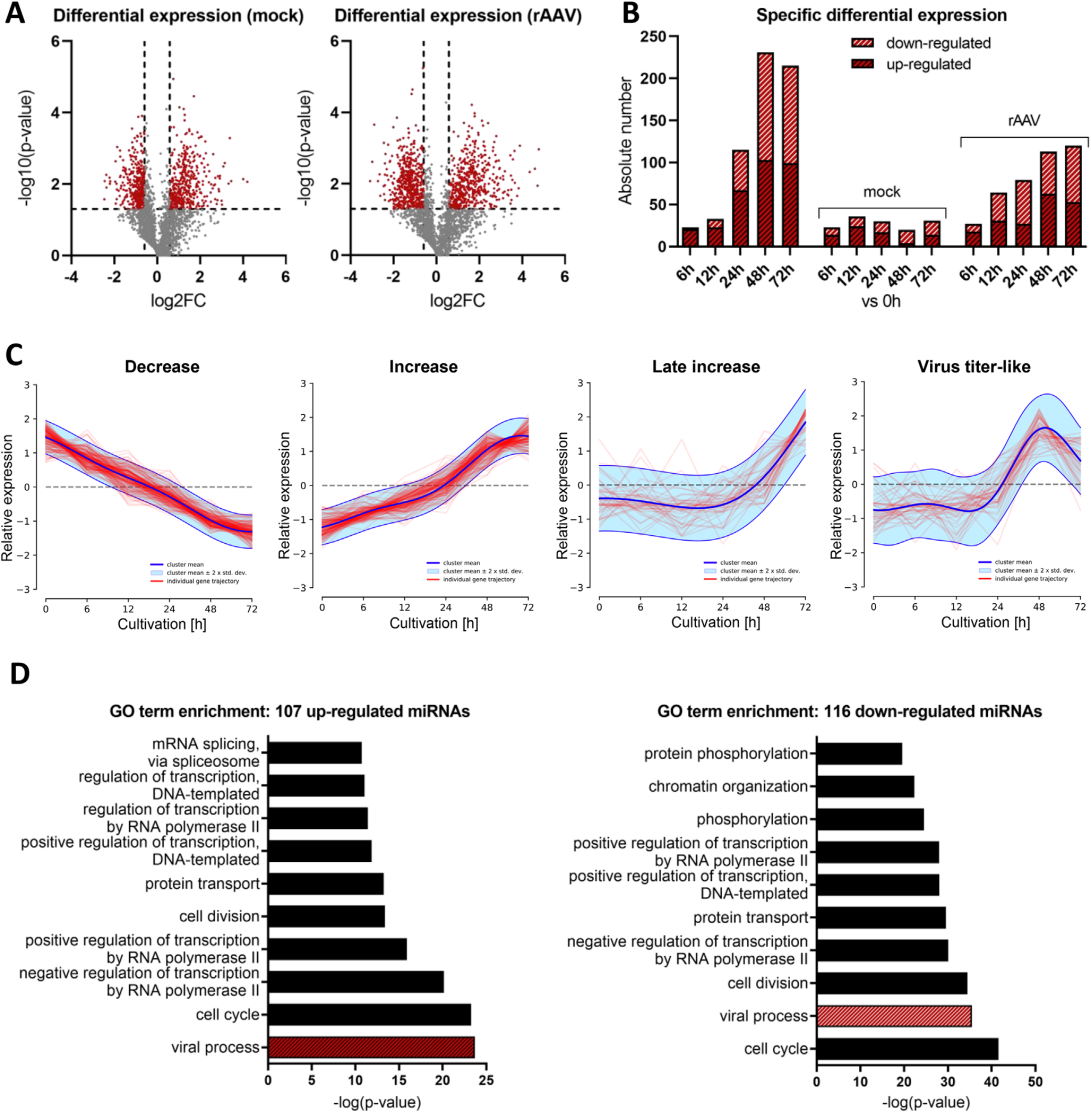
Overview of miRNA expression dynamics during mock and rAAV transfection relative to basal expression. (A) Volcano plot considering all expression events (grey and red dots) during mock (*n* = 2480) and rAAV transfection (*n* = 2551) relative to basal expression. Differential expression is considered significant (red dots, mock: *n* = 214, rAAV: *n* = 285) for a log2 fold change (log2FC) of ±0.5849 (vertical lines) and a ‐log10 *p* value of 1.3 (horizontal line). (B) Specific number of up‐ and downregulated miRNAs (difference in fold change ≥1.5, *p* value <0.05; FDR <0.1) at each sampling time point identified in both, mock or rAAV transfection relative to basal expression. (**C)** miRNA clustering according to temporal changes during rAAV transfection using the DPGP mixture model. The x and y axis represent sampling time points (in hours) and relative log2‐transformed expression values to basal expression without standardization for all biological triplicates. Red lines represent individual trajectories for each miRNA relative to basal expression, while the dark blue line indicates the mean of clustered miRNAs. In addition, the range of 95% confidence interval ± 2 SD is given in light blue. (**D)** GO term enrichment for up‐ and downregulated miRNAs relative to basal expression and associated experimentally validated target genes during the main rAAV production phase (24–72 hpT) using miRPath 4.0. The term overrepresentation in targets of the input miRNAs was evaluated using one‐sided Fisher's exact test. Significant terms in GO domain ‘biological process’ were further filtered using genes union as merging method, a *p *value threshold of <0.05 and false discovery rate (FDR) correction.

**FIGURE 4:**
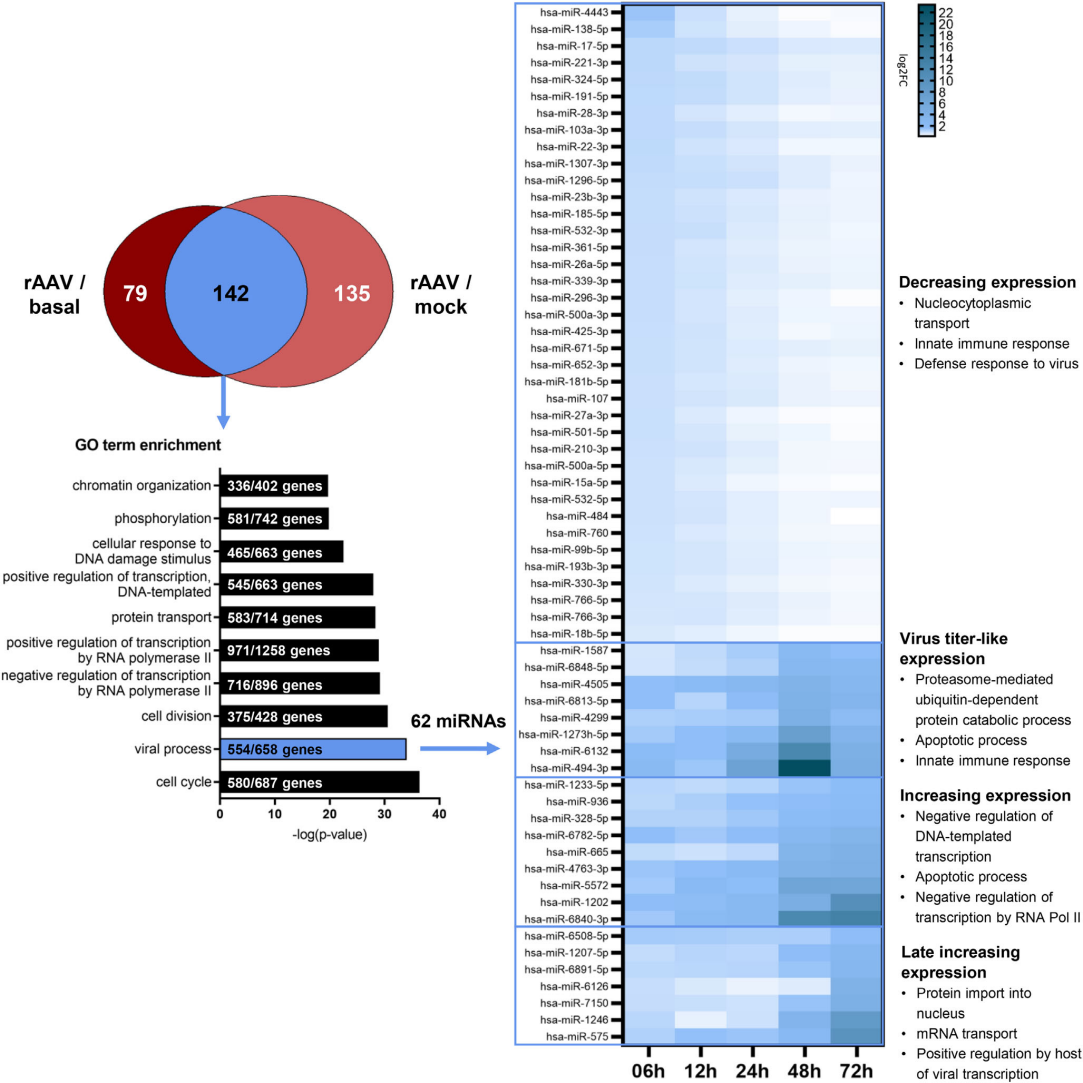
Identification of rAAV production‐specific miRNAs and associated underlying molecular mechanisms. rAAV‐specific differentially expressed miRNAs during the main rAAV production phase (24–72 hpT) relative to mock and basal expression were identified via Venn diagram analysis. The overlapping share of differentially regulated miRNAs (*n* = 142) was analyzed for GO term enrichment via miRPath 4.0 as described earlier (see Figures 2 and 3). Sixty‐two miRNAs assigned to GO term ‘viral process’ were further investigated in accordance with four specific DPGP expression patterns. Regulated target genes of miRNAs identified via miRPath 4.0 within each expression cluster were enriched for specific biological processes underlying “viral process” using DAVID.

In a second comparison, differential miRNA expression was calculated at each time point relative to basal expression at 0 h as log2 fold change for both mock‐ and rAAV‐plasmid transfected cells. Overall, 55.7% of miRNAs in mock transfection and 66.7% in rAAV transfection were differentially expressed at one or more time points compared to the 0 h baseline (Table S1). In mock‐transfected cells, a total of 757 differential expression events were observed over time, with up‐regulation reaching up to a 4.2‐fold change and down‐regulation up to −2.5‐fold (Figure 3A). In rAAV‐transfected cells, this number increased to 1020 differential expression events, with up‐regulation reaching a 4.8‐fold change and down‐regulation up to −3.1‐fold. When examining exclusive subgroups (Figure 3B), the number of differentially expressed miRNAs common to both mock‐ and rAAV‐transfected cells amounted to over 230 miRNAs at 48 hpT, whereas far fewer miRNAs were exclusively regulated in mock‐transfected cells only. Importantly, for miRNAs exclusively differentially expressed in rAAV‐producing cells, which is likely the most relevant group potentially involved in or influenced by molecular events during rAAV production, the numbers increased over time, reaching 113 at 48 hpT and 120 at 72 hpT. Using the DPGP mixture model to analyze the time course of individual miRNA expression normalized to basal expression at 0 h in rAAV‐transfected cells, 17 distinct clusters each were identified (Figure S3). Of these, 67.7% of the miRNAs were assigned to four clusters with decreasing, increasing, late increasing expression, or again following a virus titer‐like trajectory (Figure 3C). For mock transfection, four clusters with the highest numbers of miRNAs were identified, characterized by decreasing, constant, or step‐wise increasing miRNA expression, and a unique expression pattern featuring a peak in expression at 12 hpT (Figures 2 and 4).

As before, 223 miRNAs differentially expressed at the main rAAV production phase from 24 to 72 hpT in only rAAV‐producing cells were investigated for experimentally validated miRNA target genes combined with GO term enrichment analysis (Table S2B). Here again, the GO term “viral process: was highly significantly enriched in addition to terms related to transcription”, cell cycle, or cell maintenance (Figure 3D), which may again indicate an involvement of these miRNAs in molecular changes induced or influenced by rAAV production.

### 3.3 rAAV Production Specific miRNAs and Underlying Molecular Mechanisms

To further elucidate possible molecular mechanisms underlying miRNA‐regulated gene expression, differentially expressed miRNAs during the main rAAV production phase from 24 to 72 hpT were subjected to Venn diagram analysis. This analysis identified 142 miRNAs differentially expressed across both comparisons (Figure 4, Table S2C), of which 128 miRNAs were assigned to the GO term “viral process” through enrichment analyses of validated target genes. Temporal analysis of differential expression patterns associated with relevant GO terms downstream of “viral process” revealed 62 miRNAs distributed across the four distinct expression clusters described earlier (Figure 4). Table S3 provides a summary overview of the miRNA target genes along with the associated GO terms that characterize the underlying regulatory mechanisms. The first and largest cluster comprised 38 miRNAs exhibiting a progressively decreasing expression pattern and significant association with target gene GO terms such as “nucleocytoplasmic transport”, “innate immune response”, and “defense response to virus”, indicative of a cellular response to viral production. The second cluster included 8 miRNAs that displayed a virus titer‐like expression, peaking at 48 hpT. These miRNAs targeted pathways such as “proteasome‐mediated ubiquitin‐dependent protein catabolic process”, “apoptotic process”, and “innate immune response”, suggesting involvement in virus‐induced cellular pathways. In the third cluster, 9 miRNAs constantly increased in differential expression over time. These miRNAs were associated with downregulation of target genes linked to negative regulation of transcription as well as apoptotic processes, potentially reflecting a viral strategy to mitigate host transcriptional restrictions on replication. The last cluster included 7 miRNAs that exhibited increased expression in the late phase of rAAV production. These miRNAs were enriched in GO terms such as “protein import to nucleus”, “mRNA transport”, and “positive regulation by host of viral transcription”, pointing to a possible cellular antiviral response mechanism.

### 3.4 Differential snoRNA Regulation During rAAV Production

**FIGURE 5:**
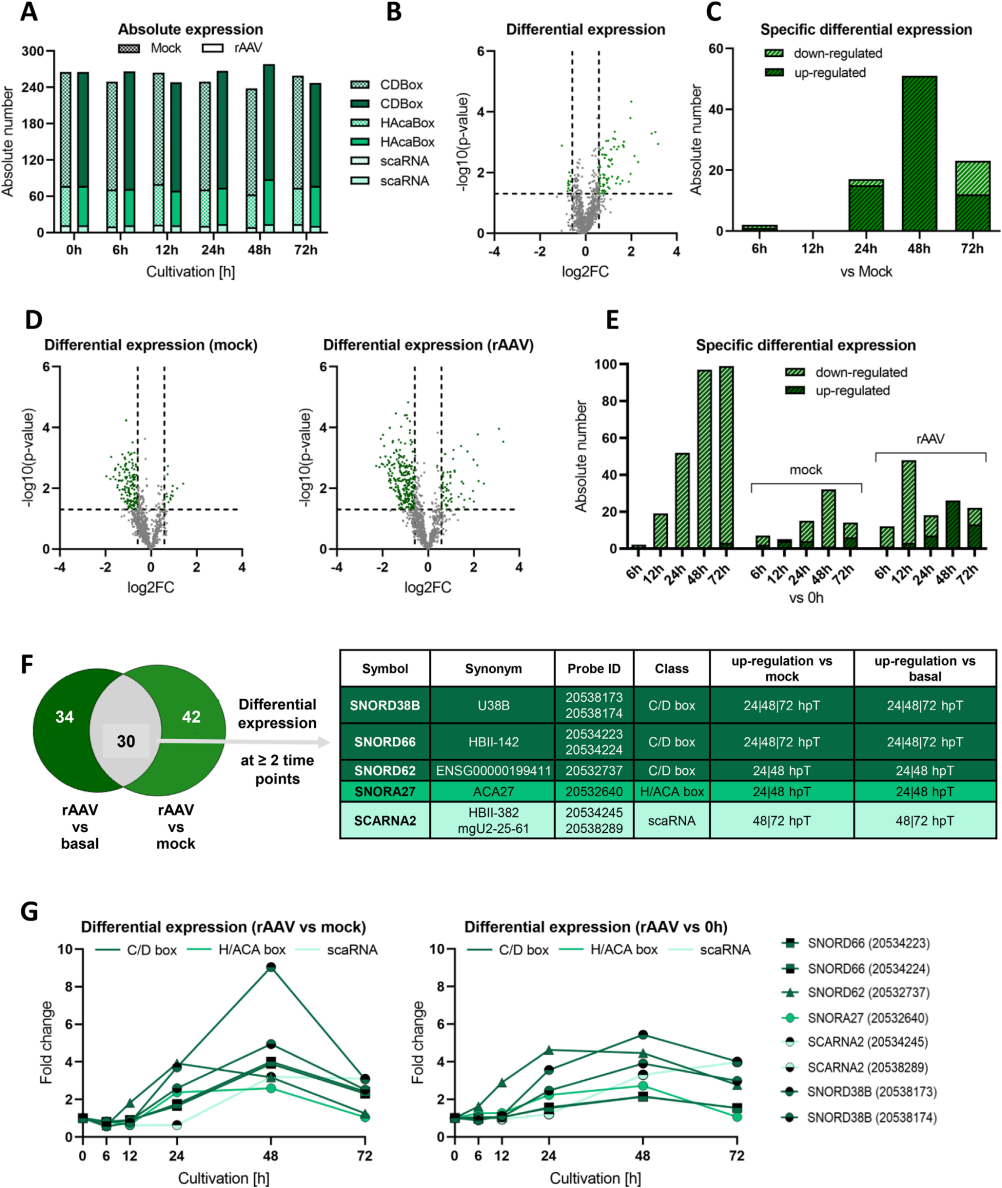
Overview of snoRNA expression dynamics during mock and rAAV transfection relative to mock transfection. (A) Absolute number of snoRNAs expressed during mock (checked) and rAAV transfection (filled) at each sampling time point. In addition, snoRNAs were categorized by their class into C/D box snoRNAs (dark green), H/ACA box snoRNAs (green), and scaRNAs (light green). (B), (D) Volcano plot considering all expression events (grey and green dots) during (B) rAAV transfection relative to mock transfection (*n* = 1428) and (D) during mock (*n* = 1025) and rAAV transfection (*n* = 1314) relative to basal expression. Differential expression is considered significant (green dots, rAAV relative to mock: *n* = 93, mock relative to basal: *n* = 350, rAAV relative to basal: *n* = 417) for a log2 fold change (log2FC) of ±0.5849 (vertical lines) and a ‐log10 *p* value of 1.3 (horizontal line). (C), (E) Specific number of up‐ and downregulated snoRNAs (difference in fold change ≥1.5, *p* value <0.05; FDR <0.1) at each sampling time point (C) relative to mock transfection or (E) identified in both, mock or rAAV transfection relative to basal expression. (F) Identification of rAAV‐specific differentially expressed snoRNAs during the main rAAV production phase (24–72 hpT) relative to mock and basal expression via Venn diagram analysis. The overlapping share of differentially regulated snoRNAs (*n* = 5) was filtered for candidates exhibiting differential expression at two or three consecutive sampling time points. Three snoRNAs (SNORD38B, SNORD66, SCARNA2) were covered with two distinct probes in the microarray analysis. (G) Analysis of rAAV‐specific differential expression pattern relative to either mock transfection or basal expression. snoRNAs were categorized by their class into C/D box snoRNAs (dark green), H/ACA box snoRNAs (green), and scaRNAs (light green).

In addition to analyzing the miRNome, we investigated the expression of small nucleolar RNAs, collectively termed the snoRNome, a field that has received limited attention, particularly regarding potential effects during rAAV production. Overall, the snoRNome exhibited lower numbers to be expressed compared to the miRNome, encompassing a total of 352 expressed snoRNAs. These were categorized into 232 C/D box snoRNAs, 100 H/ACA box snoRNAs, and 20 scaRNAs. The total number of expressed snoRNAs remained relatively stable over time (Figure 5A). In rAAV‐transfected cells, the highest expression level was observed at 48 hpT, with a total of 279 snoRNAs. In contrast, mock‐transfected cells exhibited peak expression at 0 hpT, with 267 snoRNAs (Figure 5A).

We employed a multi‐level comparative strategy to analyze differences in snoRNome expression. Comparing snoRNA expression in rAAV‐transfected cells to mock‐transfected controls, 21.3% of snoRNAs exhibited differential expression at one or more time points (Table S1). Over the analyzed time course, 93 differential expression events were identified, predominantly involving upregulation, with fold changes reaching up to 3.2‐fold (Figure 5B). The highest number of upregulated snoRNAs was observed at 48 hpT (Figure 5C). Differential snoRNA expression was next calculated at each time point relative to basal expression at 0 h for both mock‐ and rAAV‐plasmid transfected cells, where 42.9% of snoRNAs in mock transfection and 38.9% in rAAV transfection were differentially expressed at one or more time points compared to the 0 h baseline (Table S1). Notably, both mock and rAAV‐transfected cells showed significantly more down‐regulation events compared to the basal level (Figure 5D). In mock transfection, a total of 350 differential expression events were observed over the 72‐h cultivation period, with changes ranging from approximately −2‐fold downregulation to 1.4‐fold upregulation (Figure 5D). For rAAV‐transfected cells, differential expression events amounted to 417 in total, with changes of up to 3.3‐fold upregulation and −2.3‐fold downregulation. Differential expression at each sampling time point increased to almost 100 snoRNAs at 48 and 72 hpT for both mock and rAAV transfection, predominantly showing downregulation (Figure 5E). Interestingly, the highest number of down‐regulated snoRNAs exclusively in mock‐transfected cells peaked at 48 hpT. Conversely, snoRNAs uniquely differentially expressed in rAAV‐transfected cells were predominantly upregulated at 48 hpT, while peaking at 12 hpT with a predominance of downregulated snoRNAs.

In summary, a general trend of snoRNA downregulation relative to basal expression at 0 h was observed. Interestingly, this downregulation was less pronounced in rAAV‐transfected cells, where an upregulation compared to the mock control was detected. This observation points to a distinct regulatory pattern in rAAV‐transfected samples.

### 3.5 rAAV Production Specific snoRNAs With Significant Differential Expression

The role of snoRNAs in viral infection and replication remains largely unexplored, prompting us to identify specific snoRNAs likely involved in or influenced by rAAV production. A Venn diagram analysis of 106 rAAV‐specific differentially expressed snoRNAs versus basal and mock expression during the main rAAV production phase, starting at 24 hpT, revealed 30 snoRNAs differentially regulated in both comparisons (Figure 5F). Among these, 5 were identified to be differentially expressed at two or three consecutive time points during the main rAAV production phase. Relative to mock transfection, fold changes in differential expression ranged from 2.6 for SNORA27 to as high as 9‐fold for SNORD38B at 48 hpT (Figure 5G). While SNORD38B, SNORD66, and SCARNA2 exhibited a differential expression pattern closely resembling virus titer progression, SNORA27 and SNORD62 did not follow this pattern, but showing an increase in expression up to 24 hpT, followed by a decline by 72 hpT. A similar trend was observed for differential expression relative to basal levels, although with less pronounced fold changes.

Our findings suggest that specific snoRNAs, particularly SNORD38B, SNORD66, and SCARNA2, may play a role in rAAV production, as their expression patterns closely align with viral titer progression, while others, such as SNORA27 and SNORD62, exhibit distinct temporal regulation, indicating potential diverse functions in the process.

## 4 Discussion

Meeting the increasing demand for high‐dose adeno‐associated viral vectors in gene therapy requires scalable and cost‐effective production platforms. While recent advances, such as in silico modeling, stable producer cell lines, continuous manufacturing, and improved purification methods, have addressed key technical bottlenecks [29], a more foundational understanding of the cellular response to rAAV production is still needed. Multi‐omics technologies offer a powerful means to characterize the dynamic molecular changes that occur within production cells, providing insight into how viral replication, host defense mechanisms, and cellular stress responses are orchestrated at the systems level [4, 29]. Despite the growing application of omics‐based approaches, the specific roles of non‐coding RNAs, particularly miRNAs and snoRNAs, in shaping these responses remain largely unexplored. This study aims to describe the expression dynamics of these regulatory RNAs and analyze their potential influence on biological processes that encompass the cellular and viral response during rAAV production.

Throughout the cultivation and sampling process, particularly during the main phase of rAAV production (24–72 hpT), a substantial number of differentially expressed miRNAs were observed compared to mock transfection and basal expression levels at 0 h. This outcome aligns with prior evidence that miRNAs are pivotal in post‐transcriptional regulation of gene expression, impacting fundamental cellular pathways and networks [19, 47]. Analysis of these differentially expressed miRNAs during the main production phase revealed enrichment in GO terms associated with biological processes such as transcription regulation, cell cycle, and viral processes, indicating a potential role in modulating viral replication and host‐virus interactions. During rAAV production, 62 miRNAs were differentially expressed and grouped into four clusters. Thereof, 32 early downregulated miRNAs influence nucleocytoplasmic transport by targeting nucleoporins (NUPs), potentially enhancing nuclear import of viral particles, a key step in efficient rAAV transduction [48, 49, 50]. Additionally, the helper gene E4, encoded on the plasmid, interacts with NUP205, a nucleoporin regulated by differentially expressed miRNAs hsa‐miR‐107, hsa‐miR‐138‐5p, hsa‐miR‐17‐5p, hsa‐miR‐22‐3p, hsa‐miR‐500a‐5p, and hsa‐miR‐766‐3p, to support viral gene expression [51]. We further identified the GO terms “innate immune response” and “defense response to virus” as highly enriched biological processes targeted by progressively downregulated miRNAs. Previous research by Chung et al. has demonstrated that rAAV production in HEK293 cells triggers an antiviral response [5]. Their study identified differentially expressed genes and defined 107 upstream master regulators potentially modulating the observed transcriptional changes. Notably, nine of these master regulators – Cyclic GMP‐AMP Synthase (CGAS), Signal Transducer and Activator of Transcription 1 (STAT1), TIR Domain Containing Adaptor Molecule 1 (TICAM1), Interferon Regulatory Factor 3 (IRF3), Inhibitor of Nuclear Factor Kappa B Kinase Subunit Beta (IKBKB), Mitochondrial Antiviral Signaling Protein (MAVS), PML Nuclear Body Scaffold (PML), Eukaryotic Translation Initiation Factor 2 Alpha Kinase 2 (EIF2AK2), and Stimulator of Interferon Response CGAMP Interactor 1 (STING1) – were associated with immune defense mechanisms, including antiviral response, interferon production, and innate immune activation. GO term analysis performed in our study could also assign these regulators as targets of 18 distinct miRNAs, which showed decreased expression. This suggests a potential role for miRNA‐mediated regulation in modulating the host cell's immune and antiviral responses during rAAV production. The relevance of immune modulation in rAAV production has been further supported by studies demonstrating that inhibition of the Jak/STAT interferon pathway enhances rAAV yield by two‐fold [52]. Moreover, the role of miRNAs in the interferon response has been previously explored by Nazarov et al., where they identified miR‐23b‐5p and miR‐17‐5p as key regulators of interferon signaling [53]. In our study, both miRNAs were significantly downregulated, leading to the upregulation of their target genes, including Janus Kinase 1 (JAK1), BCL2 Apoptosis Regulator (BCL2), and Interferon Regulatory Factor 1 (IRF1). This observation further supports the notion that miRNA‐mediated regulation might influence canonical interferon signaling pathways, thereby impacting rAAV production dynamics.

GO analysis linked miRNAs hsa‐miR‐1273h‐5p and hsa‐miR‐494‐3p with virus titer‐like expression to ubiquitin‐mediated degradation, consistent with studies showing the ubiquitin‐proteasome system's influence on rAAV intracellular trafficking and second‐strand DNA synthesis [54, 55]. Additionally, miRNAs downregulating apoptosis, which comprises the above‐mentioned but also hsa‐miR‐4299, hsa‐miR‐4505, hsa‐miR‐6132, and hsa‐miR‐6813‐5p, may counteract p53‐dependent stress responses, facilitating viral replication [56, 57]. Lastly, miRNAs upregulated over time were all associated with transcriptional repression, reducing target gene expression. Tworig et al. demonstrated that suppressing negative transcriptional regulators promotes AAV9 production, a finding supported by our results [35]. These observations underscore the pivotal role of miRNAs in regulating rAAV production and related cellular pathways.

Notably, three miRNAs – hsa‐miR‐1207‐5p, hsa‐miR‐6126, and hsa‐miR‐6891‐5p – exhibited a late‐stage increase in differential expression and were associated with the positive regulation of viral transcription. Among predicted miRNA targets, E1A Binding Protein P300 (EP300) and SNW Domain Containing 1 (SNW1) were linked to both the activation and repression of transcription. In contrast, SMARCB1 (SWI/SNF Related BAF Chromatin Remodeling Complex Subunit B1) and SP1 Transcription Factor were uniquely associated with transcriptional activation and have been implicated in promoting viral replication. Specifically, SMARCB1 inhibition has been shown to impair antiviral responses by disrupting cellular defenses against viral infection, whereas SP1 is known to enhance viral gene transcription, as observed in herpes simplex virus [58, 59]. These findings suggest that the increased expression of these miRNAs may simultaneously enhance both pro‐ and antiviral cellular responses, highlighting a potential dual regulatory role of miRNAs in transcriptional modulation during viral infection.

Analysis of snoRNA dynamics revealed fewer differentially expressed molecules compared to miRNAs, consistent with a more specialized role of snoRNAs in site‐specific methylation and pseudouridylation of rRNAs [20]. Although less studied, snoRNAs are implicated in health and disease, with functions such as miRNA‐like activity, RNA interactions, as well as interaction with non‐canonical RNA‐binding proteins for ribosome biogenesis [26, 28, 60]. Related thereto, Liu et al. identified novel snoRNA‐mRNA interactions that influence protein secretion, highlighting the therapeutic and biotechnological relevance of snoRNAs [61]. Furthermore, Schreiber et al. identified PHD finger‐like domain protein 5A (PHF5A), which is associated with the U2 snRNP spliceosome, as a host restriction factor in rAAV replication, where gene disruption led to increased transgene expression from AAV and, additionally, might influence further steps downstream of second‐strand synthesis. Consequently, snoRNA‐mediated modifications of U2 snRNA within the spliceosome may regulate rAAV replication [62, 63]. Nonetheless, detailed roles of snoRNAs in viral replication remain underexplored, with limited studies suggesting involvement in viral processes [17, 18].

During rAAV production, five snoRNAs, SNORD38B, SNORD66, SNORD62, SNORA27, and SCARNA2, showed significant upregulation, suggesting functional relevance. Generally speaking, C/D and H/ACA box snoRNAs can activate Protein Kinase R (PKR), triggering antiviral interferon responses [64]. In particular, SCARNA2 is known to regulate DNA repair by inhibiting DNA‐dependent protein kinases, potentially modulating AAV‐induced DNA damage responses [65, 66].

This study delineates a molecular framework for characterizing the cellular response to rAAV production, with a particular focus on the regulatory roles of miRNAs and snoRNAs in host cell adaptation. While conventional multi‐omics approaches primarily emphasize differential expression at the level of individual transcripts or proteins, the analysis of non‐coding RNAs, particularly miRNAs, enables coordinated regulation of entire gene networks and signaling pathways relevant for rAAV production. In this study, we identified a substantial number of non‐coding RNAs being differentially regulated during rAAV production. However, it remains to be elucidated whether these candidates act as upstream regulators contributing to rAAV production or represent downstream consequences of the cellular stress response towards vector manufacturing in general. Notably, viral vector transfection and production inherently impose cellular burden, potentially through mechanisms such as the accumulation of reactive oxygen species, which may initiate oxidative stress and elicit damage‐associated signaling cascades [67, 68]. Yet, these insights will in the future require functional validation via transient overexpression of candidate miRNAs and snoRNAs in host cells, followed by assessment of their effects on rAAV titers. By identifying critical regulatory RNAs and associated molecular circuits, the findings offer novel targets for systematic host cell engineering using stable miRNA knockout and overexpression strategies aimed at modulating cellular responses to finally enhance rAAV yields. Collectively, a better understanding of molecular mechanisms induced by rAAV expression will pave the way for optimized bioprocessing strategies.

## Author Contributions

Kerstin Otte and Madina Burkhart conceived the study. Katrin Langenbach, Nadine Hornung, Jamie‐Ann Baiz, and Madina Burkhart performed experiments in the laboratory. Karlheinz Holzmann did the microarray expression analysis. Karlheinz Holzmann and Madina Burkhart processed the data. Madina Burkhart analyzed the data and wrote the initial manuscript. Kerstin Otte supervised the study and provided resources.

## Conflicts of Interest

The authors declare that they have no known competing financial and non‐financial interests or personal relationships that could have appeared to influence the work reported in this paper.

## Declaration of generative AI and AI‐assisted technologies in the writing process

During the preparation of this work, the authors used ChatGPT 4o in a limited manner to improve readability and language of the manuscript. The authors reviewed and edited the content and take full responsibility for the content of the published article.

## Supporting information




**Supporting File 1**: biot70092‐sup‐0001‐FigureS1.pdf

**Supporting File 2**: biot70092‐sup‐0002‐FigureS2.pdf

**Supporting File 3**: biot70092‐sup‐0003‐FigureS3.pdf

**Supporting File 4**: biot70092‐sup‐0004‐FigureS4.pdf

**Supporting File 5**: biot70092‐sup‐0005‐TableS1.pdf

**Supporting File 6**: biot70092‐sup‐0006‐TableS2A.pdf

**Supporting File 7**: biot70092‐sup‐0007‐TableS2B.pdf

**Supporting File 8**: biot70092‐sup‐0008‐TableS2C.pdf

**Supporting File 9**: biot70092‐sup‐0009‐TableS3.xlsx

## Data Availability

The data discussed in this publication have been deposited in NCBI's Gene Expression Omnibus and are accessible through GEO Series accession number GSE289823 (https://www.ncbi.nlm.nih.gov/geo/query/acc.cgi?acc = GSE289823).

## References

[1] R. J. Samulski and N. Muzyczka , “AAV‐Mediated Gene Therapy for Research and Therapeutic Purposes,” Annual Review of Virology 1, no. 1 (2014): 427–451, 10.1146/annurev-virology-031413-085355. 26958729

[2] D. Wang , P. W. L. Tai , and G. Gao , “Adeno‐Associated Virus Vector as a Platform for Gene Therapy Delivery,” Nature Reviews Drug Discovery 18, no. 5 (2019): 358–378, 10.1038/s41573-019-0012-9. 30710128 PMC6927556

[3] J. C. Grieger and R. J. Samulski , “Adeno‐Associated Virus Vectorology, Manufacturing, and Clinical Applications,” Methods in Enzymology 507 (2012): 229–254, 10.1016/B978-0-12-386509-0.00012-0. 22365777

[4] J. Smith , J. Grieger , and R. J. Samulski , “Overcoming Bottlenecks in AAV Manufacturing for Gene Therapy,” Cell and Gene Therapy Insights 4, no. 8 (2018): 815–825, 10.18609/cgti.2018.083.

[5] C. H. Chung , C. M. Murphy , V. P. Wingate , et al., “Production of rAAV by Plasmid Transfection Induces Antiviral and Inflammatory Responses in Suspension HEK293 Cells,” Molecular Therapy Methods and Clinical Development 28 (2023): 272–283, 10.1016/j.omtm.2023.01.002. 36819978 PMC9937832

[6] Y. Wang , Q. Fu , S. Y. Park , et al., “Decoding Cellular Mechanism of Recombinant Adeno‐Associated Virus (rAAV) and Engineering Host‐Cell Factories Toward Intensified Viral Vector Manufacturing,” Biotechnology Advances 71 (2024): 108322, 10.1016/j.biotechadv.2024.108322. 38336188

[7] M. H. Orzalli and D. M. Knipe , “Cellular Sensing of Viral DNA and Viral Evasion Mechanisms,” Annual Review of Microbiology 68 (2014): 477–492, 10.1146/annurev-micro-091313-103409. PMC4348004 25002095

[8] P. Ladiwala , N. Ndahiro , P. Hauk , et al., “Unraveling Cytotoxicity in HEK293 Cells During Recombinant AAV Production for Gene Therapy Applications,” Biotechnology Journal 20, no. 3 (2025): 202400501, 10.1002/biot.202400501. 40079705

[9] M. Schmidt , S. Afione , and R. M. Kotin , “Adeno‐Associated Virus Type 2 Rep78 Induces Apoptosis Through Caspase Activation Independently of p53,” Journal of Virology 74, no. 20 (2000): 9441–9450, 10.1128/jvi.74.20.9441-9450.2000. 11000213 PMC112373

[10] D. Brockmann and H. Esche , “The Multifunctional Role of E1A in the Transcriptional Regulation of CREB/CBP‐Dependent Target Genes,” in Adenoviruses: Model and Vectors in Virus‐Host Interactions: Virion‐Structure, Viral Replication and Host‐Cell Interactions, ed W. Doerfler and P. Böhm (Springer, 2003), 97–129, 10.1007/978-3-662-05597-7_4. 12747548

[11] P. Fax , K. S. Lipinski , H. Esche , and D. Brockmann , “cAMP‐Independent Activation of the Adenovirus Type 12 E2 Promoter Correlates With the Recruitment of CREB‐1/ATF‐1, E1A12S, and CBP to the E2‐CRE,” Journal of Biological Chemistry 275, no. 12 (2000): 8911–8920, 10.1074/jbc.275.12.8911. 10722738

[12] M. Debbas and E. White , “Wild‐Type p53 Mediates Apoptosis by E1A, which is inhibited by E1B,” Genes & Development 7, no. 4 (1993): 546–554, 10.1101/gad.7.4.546. 8384580

[13] R. A. Schwartz , J. A. Palacios , G. D. Cassell , S. Adam , M. Giacca , and M. D. Weitzman , “The Mre11/Rad50/Nbs1 Complex Limits Adeno‐Associated Virus Transduction and Replication,” Journal of Virology 81, no. 23 (2007): 12936–12945, 10.1128/JVI.01523-07. 17898048 PMC2169118

[14] E. Girardi , P. López , and S. Pfeffer , “On the Importance of Host MicroRNAs during Viral Infection,” Frontiers in Genetics 9 (2018): 439, 10.3389/fgene.2018.00439. 30333857 PMC6176045

[15] M. G. Barbu , C. E. Condrat , D. C. Thompson , et al., “MicroRNA Involvement in Signaling Pathways during Viral Infection,” Frontiers in Cell and Developmental Biology 8 (2020): 143, 10.3389/fcell.2020.00143. 32211411 PMC7075948

[16] W. Rozek , M. Kwasnik , W. Socha , B. Czech , and J. Rola , “Profiling of snoRNAs in Exosomes Secreted From Cells Infected With Influenza A Virus,” International Journal of Molecular Sciences 26, no. 1 (2025): 12, 10.3390/ijms26010012. PMC11720657 39795871

[17] S. Stamm and J. S. Lodmell , “C/D Box snoRNAs in Viral Infections: RNA Viruses Use Old Dogs for New Tricks,” Non‐Coding RNA Research 4, no. 2 (2019): 46–53, 10.1016/j.ncrna.2019.02.001. 31193534 PMC6533054

[18] J. L. Murray , J. Sheng , and D. H. Rubin , “A Role for H/ACA and C/D Small Nucleolar RNAs in Viral Replication,” Molecular Biotechnology 56, no. 5 (2014): 429–437, 10.1007/s12033-013-9730-0. 24477674 PMC7090452

[19] A. M. Gurtan and P. A. Sharp , “The Role of miRNAs in Regulating Gene Expression Networks,” Journal of Molecular Biology 425, no. 19 (2013): 3582–3600, 10.1016/j.jmb.2013.03.007. 23500488 PMC3757117

[20] C. L. Holley and V. K. Topkara , “An Introduction to Small Non‐Coding RNAs: MiRNA and snoRNA,” Cardiovascular Drugs and Therapy 25, no. 2 (2011): 151–159, 10.1007/s10557-011-6290-z. 21573765

[21] Z. Huang , Y. Du , J. Wen , B. Lu , and Y. Zhao , “snoRNAs: Functions and Mechanisms in Biological Processes, and Roles in Tumor Pathophysiology,” Cell Death Discovery 8, no. 1 (2022): 259, 10.1038/s41420-022-01056-8. 35552378 PMC9098889

[22] K. I. Zhou , C. V Pecot , and C. L. Holley , “2′‐ O ‐methylation (Nm) in RNA: Progress, Challenges, and Future Directions,” RNA 30, no. 5 (2024): 570–582, 10.1261/rna.079970.124. 38531653 PMC11019748

[23] E. K. Borchardt , N. M. Martinez , and W. V. Gilbert , “Regulation and Function of RNA Pseudouridylation in Human Cells,” Annual Review of Genetics 54 (2020): 309–336, 10.1146/annurev-genet-112618-043830. PMC8007080 32870730

[24] P. Richard , X. Darzacq , E. Bertrand , B. E. Jády , C. Verheggen , and T. Kiss , “A Common Sequence Motif Determines the Cajal Body‐Specific Localization of Box H/ACA scaRNAs,” EMBO Journal 22, no. 16 (2003): 4283–4293, 10.1093/emboj/cdg394. 12912925 PMC175784

[25] J. O'Brien , H. Hayder , Y. Zayed , and C. Peng , “Overview of MicroRNA Biogenesis, Mechanisms of Actions, and Circulation,” Frontiers in Endocrinology 9 (2018): 402, 10.3389/fendo.2018.00402. 30123182 PMC6085463

[26] T. Bratkovič , J. Božič , and B. Rogelj , “Functional Diversity of Small Nucleolar RNAs,” Nucleic Acids Research 48, no. 4 (2020): 1627–1651, 10.1093/nar/gkz1140. 31828325 PMC7038934

[27] X. Wang and W. Zhao , “Research Progress on miRNAs Function in the Interaction Between human Infectious Viruses and Hosts: A Review,” Biomolecules & Biomedicine 24, no. 6 (2024): 1452–1462, 10.17305/bb.2024.10821. 39101759 PMC11496870

[28] W. Chauhan , S. SJ , S. Kafle , and R. Zennadi , “SnoRNAs: Exploring Their Implication in Human Diseases,” International Journal of Molecular Sciences 25, no. 13 (2024): 7202, 10.3390/ijms25137202. 39000310 PMC11240930

[29] Q. Fu , A. Polanco , Y. S. Lee , and S. Yoon , “Critical Challenges and Advances in Recombinant Adeno‐Associated Virus (rAAV) Biomanufacturing,” Biotechnology and Bioengineering 120, no. 9 (2023): 2601–2621, 10.1002/bit.28412. 37126355

[30] M. Pistek , C.‐I. Kahlig , and M. Hackl , et al., “Comprehensive mRNA‐Sequencing‐Based Characterization of Three HEK‐293 Cell Lines During an rAAV Production Process for Gene Therapy Applications,” Biotechnology Journal 18, no. 8 (2023): 2200513, 10.1002/biot.202200513. 37191240

[31] Y. Wang , Q. Fu , Y. S. Lee , S. Sha , and S. Yoon , “Transcriptomic Features Reveal Molecular Signatures Associated With Recombinant Adeno‐Associated Virus Production in HEK293 Cells,” Biotechnology Progress 39, no. 4 (2023): 3346, 10.1002/btpr.3346. 37130170

[32] Y.‐C. Lin , M. Lu , W. Cai , and W.‐S. Hu , “Comparative Transcriptomic and Proteomic Kinetic Analysis of Adeno‐Associated Virus Production Systems,” Applied Microbiology and Biotechnology 108, no. 1 (2024): 385, 10.1007/s00253-024-13203-5. 38896252 PMC11186941

[33] L. Strasser , S. Boi , F. Guapo , et al., “Proteomic Landscape of Adeno‐Associated Virus (AAV)‐Producing HEK293 Cells,” International Journal of Molecular Sciences 22, no. 21 (2021): 11499, 10.3390/ijms222111499. 34768929 PMC8584267

[34] M. Lu , Z. Lee , and W.‐S. Hu , “Multi‐Omics Kinetic Analysis of Recombinant Adeno‐Associated Virus Production by Plasmid Transfection of HEK293 Cells,” Biotechnology Progress 40, no. 2 (2024): 3428, 10.1002/btpr.3428. 38289617

[35] J. Tworig , F. Grafton , K. Fisher , M. Hörer , C. A. Reid , and M. A. Mandegar , “Transcriptomics‐Informed Pharmacology Identifies Epigenetic and Cell Cycle Regulators That Enhance AAV Production,” Molecular Therapy—Methods & Clinical Development 32, no. 4 (2024): 101384, 10.1016/j.omtm.2024.101384. 39687728 PMC11647610

[36] L. Zehetner , D. Széliová , B. Kraus , J. A. Hernandez Bort , and J. Zanghellini , “Multi‐omics driven genome‐scale metabolic modeling improves viral vector yield in HEK293,” Metabolic Engineering, 91, (2025): 103–118, 10.1016/j.ymben.2025.03.011. 40220853

[37] R. A. Irizarry , B. Hobbs , F. Collin , et al., “Exploration, Normalization, and Summaries of High Density Oligonucleotide Array Probe Level Data,” Biostatistics (Oxford, England) 4, no. 2 (2003): 249–264, 10.1093/biostatistics/4.2.249. 12925520

[38] N. Raab , N. Zeh , P. Schlossbauer , et al., “A Blueprint From Nature: MiRNome Comparison of Plasma Cells and CHO Cells to Optimize Therapeutic Antibody Production,” New Biotechnology 66 (2022): 79–88, 10.1016/j.nbt.2021.10.005. 34710621

[39] Y. Benjamini and Y. Hochberg , “Controlling the False Discovery Rate: A Practical and Powerful Approach to Multiple Testing,” Journal of the Royal Statistical Society: Series B (Methodological) 57, no. 1 (1995): 289–300, 10.1111/j.2517-6161.1995.tb02031.x.

[40] R. Edgar , M. Domrachev , and A. E. Lash , “Gene Expression Omnibus: NCBI Gene Expression and Hybridization Array Data Repository,” Nucleic Acids Research 30, no. 1 (2002): 207–210, 10.1093/nar/30.1.207. 11752295 PMC99122

[41] I. C. McDowell , D. Manandhar , C. M. Vockley , A. K. Schmid , T. E. Reddy , and B. E. Engelhardt , “Clustering Gene Expression Time Series Data Using an Infinite Gaussian Process Mixture Model,” PLoS Computational Biology 14, no. 1 (2018): 1005896, 10.1371/journal.pcbi.1005896. PMC5786324 29337990

[42] S. Tastsoglou , G. Skoufos , M. Miliotis , et al., “DIANA‐miRPath v4.0: Expanding Target‐Based miRNA Functional Analysis in Cell‐Type and Tissue Contexts,” Nucleic Acids Research 51 (2023): W154–W159, 10.1093/nar/gkad431. 37260078 PMC10320185

[43] D. Karagkouni , M. D. Paraskevopoulou , S. Chatzopoulos , et al., “DIANA‐TarBase v8: A Decade‐Long Collection of Experimentally Supported miRNA–Gene Interactions,” Nucleic Acids Research 46, no. D1 (2018): D239–D245, 10.1093/nar/gkx1141. 29156006 PMC5753203

[44] H.‐Y. Huang , Y.‐C.‐D. Lin , S. Cui , et al., “miRTarBase Update 2022: An Informative Resource for Experimentally Validated miRNA–Target Interactions,” Nucleic Acids Research 50, no. D1 (2022): D222–D230, 10.1093/nar/gkab1079. 34850920 PMC8728135

[45] B. T. Sherman , M. Hao , J. Qiu , et al., “DAVID: A Web Server for Functional Enrichment Analysis and Functional Annotation of Gene Lists (2021 Update),” Nucleic Acids Research 50 (2022): W216–W221, 10.1093/nar/gkac194. 35325185 PMC9252805

[46] G. Stelzer , N. Rosen , I. Plaschkes , et al., “The GeneCards Suite: From Gene Data Mining to Disease Genome Sequence Analyses,” Current Protocols in Bioinformatics 54 (2016): 1.30.1–1.30.33, 10.1002/cpbi.5. 27322403

[47] J. Shu , B. V. R. e. Silva , T. Gao , Z. Xu , and J. Cui , “Dynamic and Modularized MicroRNA Regulation and Its Implication in Human Cancers,” Scientific Reports 7, no. 1 (2017): 13356, 10.1038/s41598-017-13470-5. 29042600 PMC5645395

[48] M. Porwal , S. Cohen , K. Snoussi , et al., “Parvoviruses Cause Nuclear Envelope Breakdown by Activating Key Enzymes of Mitosis,” PLoS Pathogens 9, no. 10 (2013): 1003671, 10.1371/journal.ppat.1003671. PMC3814971 24204256

[49] S. C. Nicolson and R. J. Samulski , “Recombinant Adeno‐Associated Virus Utilizes Host Cell Nuclear Import Machinery To Enter the Nucleus,” Journal of Virology 88, no. 8 (2014): 4132–4144, 10.1128/jvi.02660-13. 24478436 PMC3993727

[50] L. A. De Jesús‐González , S. Palacios‐Rápalo , J. M. Reyes‐Ruiz , et al., “The Nuclear Pore Complex Is a Key Target of Viral Proteases to Promote Viral Replication,” Viruses. 13, no. 4 (2021): 706, 10.3390/v13040706. 33921849 PMC8073804

[51] Y. Lu , T. J. Kucharski , I. Gamache , P. Blanchette , P. E. Branton , and J. G. Teodoro , “Interaction of Adenovirus Type 5 E4orf4 With the Nuclear Pore Subunit Nup205 Is Required for Proper Viral Gene Expression,” Journal of Virology 88, no. 22 (2014): 13249–13259, 10.1128/JVI.00933-14. 25210169 PMC4249058

[52] C.‐I. Kahlig , S. Moser , L. Micutkova , J. Grillari , B. Kraus , and J. A. Hernandez Bort , “Enhancement of rAAV Titers Via Inhibition of the Interferon Signaling Cascade in Transfected HEK293 Suspension Cultures,” Biotechnology Journal 19, no. 5 (2024): 2300672, 10.1002/biot.202300672. 38719621

[53] P. V Nazarov , S. E. Reinsbach , A. Muller , et al., “Interplay of microRNAs, Transcription Factors and Target Genes: Linking Dynamic Expression Changes to Function,” Nucleic Acids Research 41, no. 5 (2013): 2817–2831, 10.1093/nar/gks1471. 23335783 PMC3597666

[54] S.‐C. Tang , A. Sambanis , and E. Sibley , “Proteasome Modulating Agents Induce rAAV2‐Mediated Transgene Expression in Human Intestinal Epithelial Cells,” Biochemical and Biophysical Research Communications 331, no. 4 (2005): 1392–1400, 10.1016/j.bbrc.2005.03.245. 15883029

[55] K. M. Valerdi , A. Hage , S. van Tol , R. Rajsbaum , and M. I. Giraldo , “The Role of the Host Ubiquitin System in Promoting Replication of Emergent Viruses,” Viruses. 13, no. 3 (2021): 369, 10.3390/v13030369. 33652634 PMC7996891

[56] M. L. Hirsch , B. M. Fagan , R. Dumitru , et al., “Viral Single‐Strand DNA Induces p53‐Dependent Apoptosis in Human Embryonic Stem Cells,” PLoS ONE 6, no. 11 (2011): 27520, 10.1371/journal.pone.0027520. PMC3219675 22114676

[57] L. Abaandou , D. Quan , and J. Shiloach , “Affecting HEK293 Cell Growth and Production Performance by Modifying the Expression of Specific Genes,” Cells 10, no. 7 (2021): 10, 10.3390/cells10071667. PMC8304725 34359846

[58] K. Cui , P. Tailor , H. Liu , X. Chen , K. Ozato , and K. Zhao , “The Chromatin‐Remodeling BAF Complex Mediates Cellular Antiviral Activities by Promoter Priming,” Molecular and Cellular Biology 24, no. 10 (2004): 4476–4486, 10.1128/MCB.24.10.4476-4486.2004. 15121865 PMC400460

[59] C. N. Sodroski , H. S. Oh , S.‐F. Chou , and D. M. Knipe , “Sp1 Facilitates Continued HSV‐1 Gene Expression in the Absence of Key Viral Transactivators,” MBio 15, no. 3 (2024): 0347923, 10.1128/mbio.03479-23. PMC10936440 38349188

[60] M. S. Scott and M. Ono , “From snoRNA to miRNA: Dual Function Regulatory Non‐Coding RNAs,” Biochimie 93, no. 11 (2011): 1987–1992, 10.1016/j.biochi.2011.05.026. 21664409 PMC3476530

[61] B. Liu , T. Wu , B. A. Miao , et al., “snoRNA‐Facilitated Protein Secretion Revealed by Transcriptome‐Wide snoRNA Target Identification,” Cell 188, no. 2 (2025): 465–483. e22, 10.1016/j.cell.2024.10.046. 39579764 PMC11761385

[62] C. A. Schreiber , T. Sakuma , Y. Izumiya , et al., “An siRNA Screen Identifies the U2 snRNP Spliceosome as a Host Restriction Factor for Recombinant Adeno‐Associated Viruses,” PLoS Pathogens 11, no. 8 (2015): 1–25, 10.1371/journal.ppat.1005082. PMC4526370 26244496

[63] Y. T. Yu , M. D. Shu , A. Narayanan , R. M. Terns , M. P. Terns , and J. A. Steitz , “Internal Modification of U2 Small Nuclear (snRNA) Occurs in Nucleoli of Xenopus Oocytes,” Journal of Cell Biology 152, no. 6 (2001): 1279–1288, 10.1083/jcb.152.6.1279. 11257127 PMC2199211

[64] O. A. Youssef , S. A. Safran , T. Nakamura , D. A. Nix , G. S. Hotamisligil , and B. L. Bass , “Potential Role for snoRNAs in PKR Activation During Metabolic Stress,” Proceedings of the National Academy of Sciences of the United States of America 112, no. 16 (2015): 5023–5028, 10.1073/pnas.1424044112. 25848059 PMC4413318

[65] R. A. Schwartz , C. T. Carson , C. Schuberth , and M. D. Weitzman , “Adeno‐Associated Virus Replication Induces a DNA Damage Response Coordinated by DNA‐Dependent Protein Kinase,” Journal of Virology 83, no. 12 (2009): 6269–6278, 10.1128/JVI.00318-09. 19339345 PMC2687378

[66] S. Bergstrand , E. M. O'Brien , C. Coucoravas , et al., “Small Cajal Body‐Associated RNA 2 (scaRNA2) Regulates DNA Repair Pathway Choice by Inhibiting DNA‐PK,” Nature Communications 13, no. 1 (2022): 1015, 10.1038/s41467-022-28646-5. PMC8866460 35197472

[67] M. E. H. Kayesh , M. Kohara , and K. Tsukiyama‐Kohara , “Effects of Oxidative Stress on Viral Infections: An Overview,” NPJ Viruses 3 (2025): 27, 10.1038/s44298-025-00110-3. 40295852 PMC11993764

[68] G. Zhao , P. Zhao , Y. Wang , et al., “GAPDH Suppresses Adenovirus‐Induced Oxidative Stress and Enables a Superfast Production of Recombinant Adenovirus,” Genes & Diseases 11, no. 6 (2024): 101344, 10.1016/j.gendis.2024.101344. 39188753 PMC11345542

