# Supplementary figures and images for: Unveiling Small Non‐Coding RNA Dynamics During Recombinant Adeno‐Associated Virus Production
Source: Biotechnol J. 2025 Aug 6;20(8):e70092. doi: 10.1002/biot.70092 (PMC12329270; doi:10.1002/biot.70092)

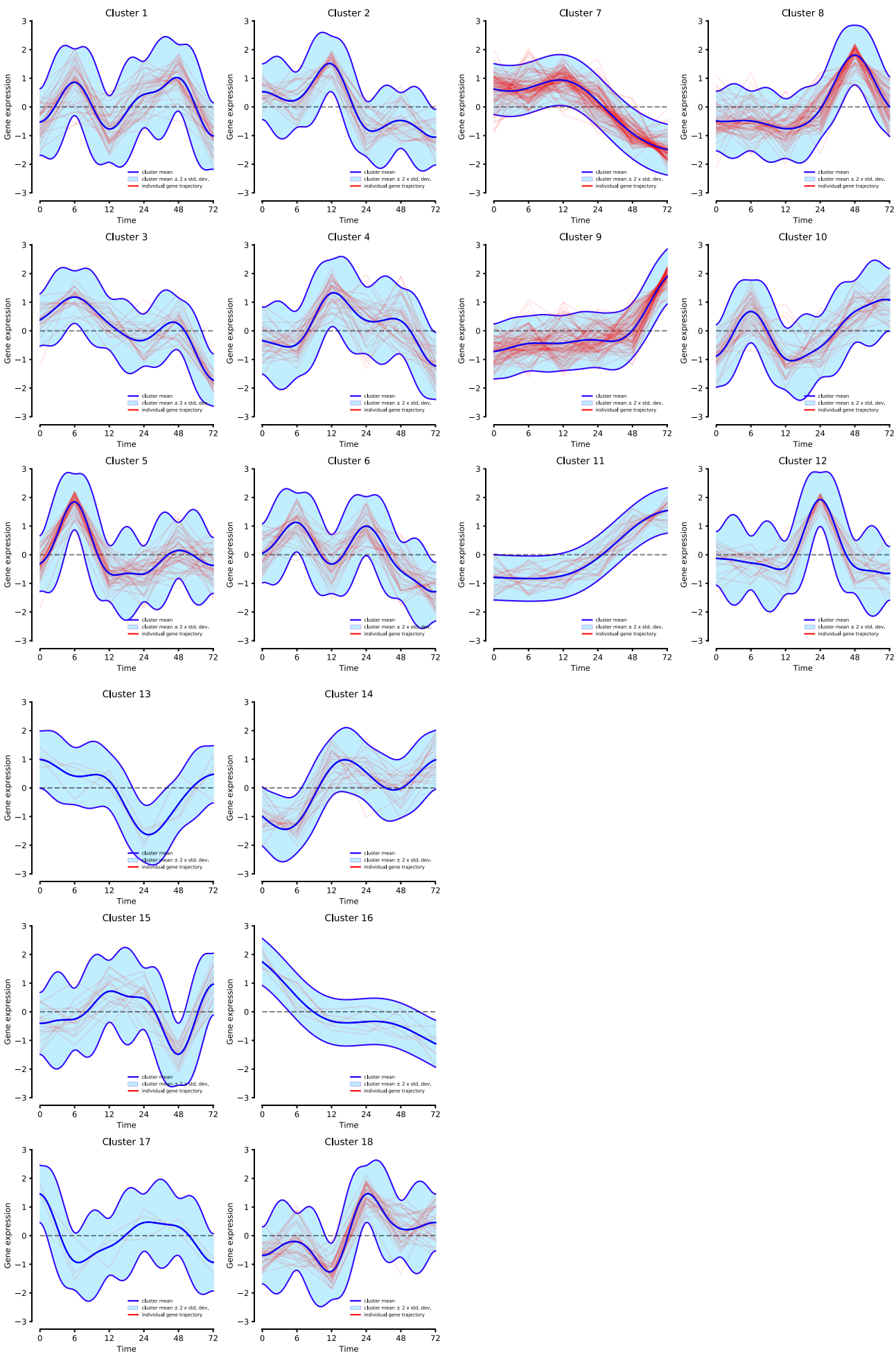

Supplement: Supplementary file 1 — Supporting File 1: biot70092‐sup‐0001‐FigureS1.pdf [file BIOT-20-e70092-s005.pdf]

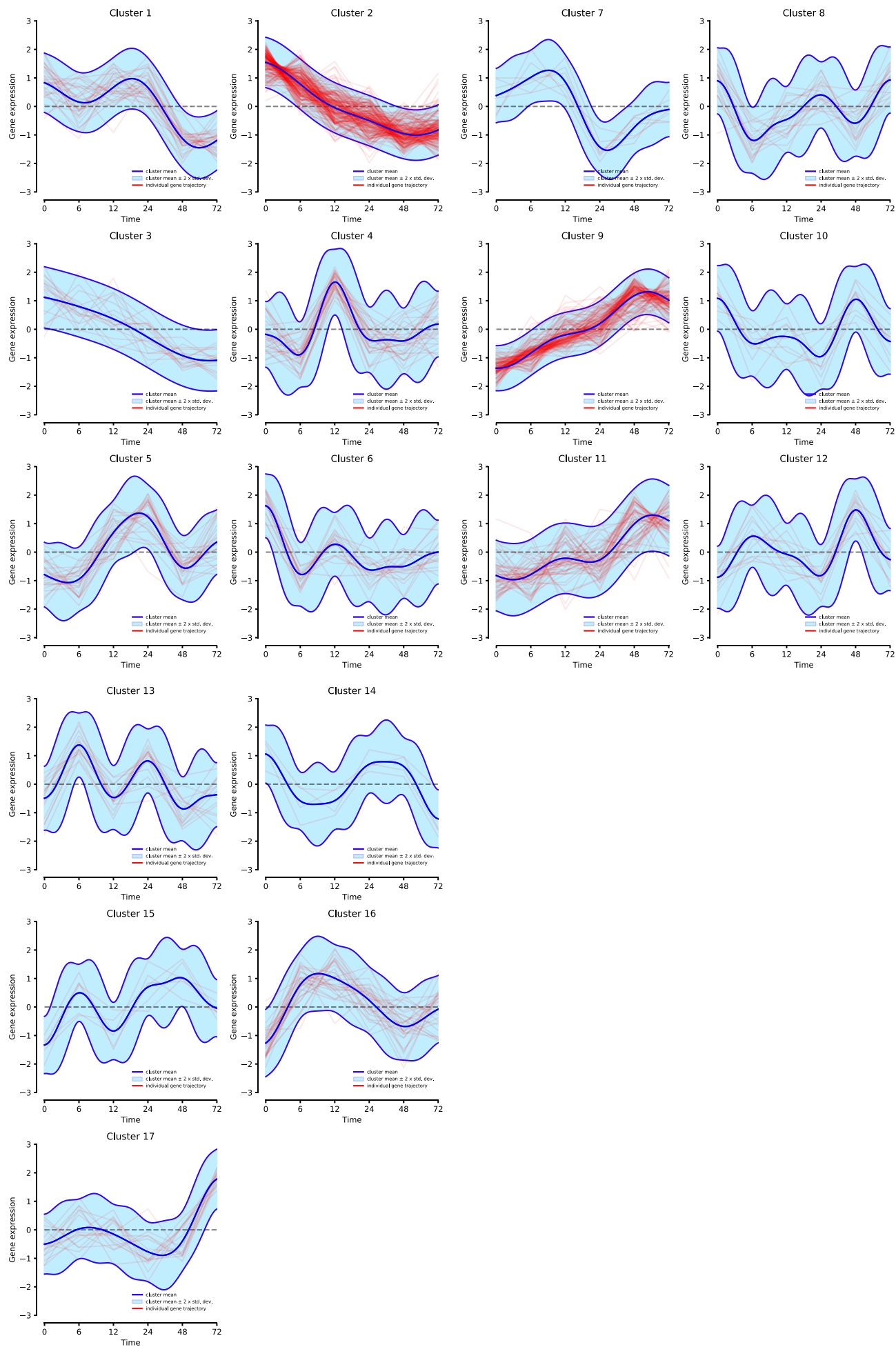

Supplement: Supplementary file 2 — Supporting File 2: biot70092‐sup‐0002‐FigureS2.pdf [file BIOT-20-e70092-s006.pdf]

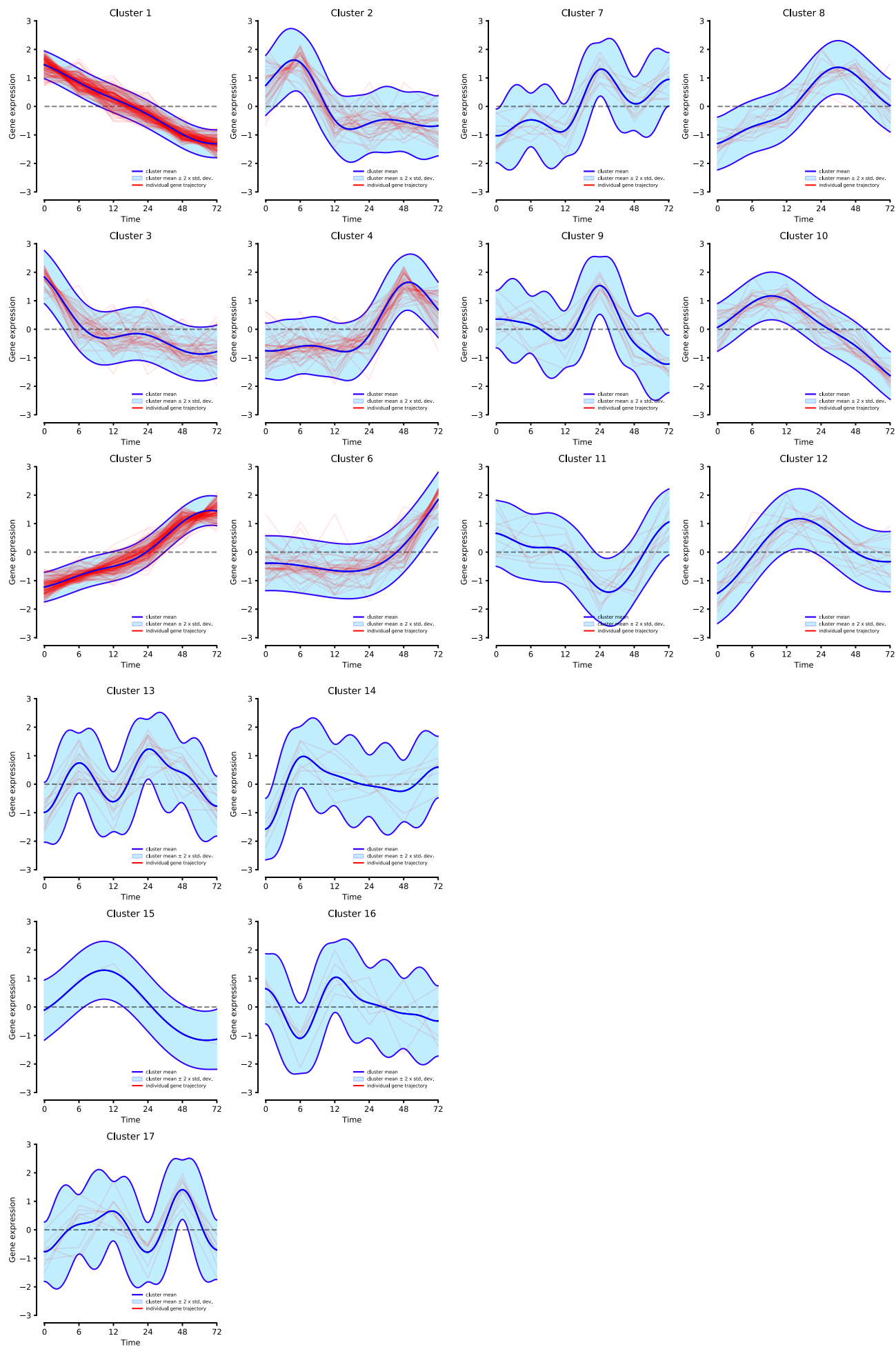

Supplement: Supplementary file 3 — Supporting File 3: biot70092‐sup‐0003‐FigureS3.pdf [file BIOT-20-e70092-s001.pdf]

**Decrease**

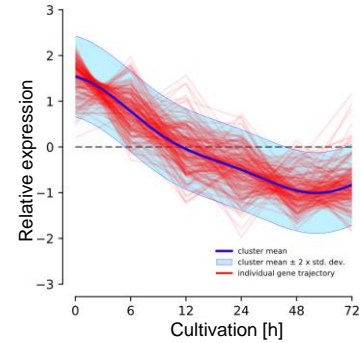

**Mock-specific response**

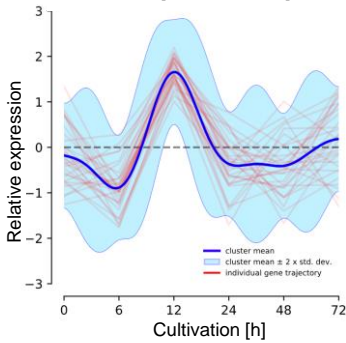

**Increase**

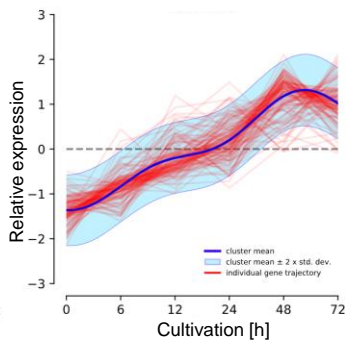

**Increase**

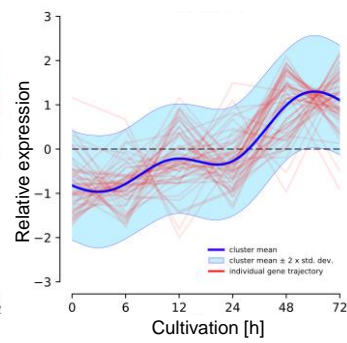

Supplement: Supplementary file 4 — Supporting File 4: biot70092‐sup‐0004‐FigureS4.pdf [file BIOT-20-e70092-s002.pdf]
