# Supplementary material for: Unveiling Small Non‐Coding RNA Dynamics During Recombinant Adeno‐Associated Virus Production
Source: Biotechnol J. 2025 Aug 6;20(8):e70092. doi: 10.1002/biot.70092 (PMC12329270; doi:10.1002/biot.70092)
Supplement: Supplementary file 5 — Supporting File 5: biot70092‐sup‐0005‐TableS1.pdf [file BIOT-20-e70092-s008.docx]

|  | **miRNome** | | **snoRNome** | |
| --- | --- | --- | --- | --- |
|  | rAAV | Mock | rAAV | Mock |
| Total number expressed at any time | 636 | | 352 | |
|  | 613 | 591 | 343 | 324 |
| Differentially expressed at any time  (vs mock) | 304  (49.6%) | - | 73  (21.3%) | - |
| Differentially expressed at any time  (vs 0 h) | 409  (66.7%) | 329  (55.7%) | 137  (38.9%) | 139  (42.9%) |
